# Medical Gaslighting and Lyme Disease: The Patient Experience

**DOI:** 10.3390/healthcare12010078

**Published:** 2023-12-29

**Authors:** Jennifer L. Fagen, Jeremy A. Shelton, Jenna Luché-Thayer

**Affiliations:** 1 Department of Sociology, Social Work, and Criminal Justice, Lamar University, P.O. Box 10026, Beaumont, TX 77710, USA; 2 Department of Psychology, Lamar University, P.O. Box 10036, Beaumont, TX 77710, USA; jashelton@lamar.edu; 3 Independent Researcher, Vero Beach, FL 32963, USA; jennaluche@gmail.com

**Keywords:** Lyme disease, chronic Lyme disease, medical gaslighting, contested illness

## Abstract

Even though there are approximately half a million new cases of Lyme disease in the US annually, according to the CDC, it is often undiagnosed or misdiagnosed, which can result in a chronic, multisystemic condition. Lyme disease is a recognized public health threat and is a designated “notifiable disease”. As such, Lyme disease is mandated to be reported by the CDC. Despite this, both acute and chronic Lyme disease (CLD) have been relegated to the category of “contested illnesses”, which can lead to medical gaslighting. By analyzing results from an online survey of respondents with Lyme disease (n = 986), we elucidate the lived experiences of people who have been pushed to the margins of the medical system by having their symptoms attributed to mental illness, anxiety, stress, and aging. Further, respondents have had their blood tests and erythema migrans (EM) rashes discounted and were told that CLD simply does not exist. As a result, a series of fruitless consultations often result in the delay of a correct diagnosis, which has deleterious consequences. This is the first study that addresses an extensive range of gaslighting techniques experienced by this patient population.

## 1. Introduction

Lyme borreliosis (Lyme disease) is caused by the spirochete bacteria *Borrelia burgdorferi* sensu lato. It is the most common tick-borne disease and the fastest growing vector-borne disease in the United States. As delineated in the following sections, Lyme disease can cause anything from flu-like symptoms to severe disabilities and death [1]. People with chronic Lyme disease (or CLD) are more likely to manifest the most disabling symptoms. Once the *Borrelia* spirochetes are disseminated throughout the body, they can affect muscles, joints, organs, and the central nervous system, breaching the blood–brain barrier [2]. It can also be transferred to the fetus during pregnancy [3,4,5]. Lyme-carrying ticks have been found in all 50 US states [6] and are carried by deer, mice, squirrels, rabbits, dogs, and cats, to name a few.

According to the Centers for Disease Control and Prevention (CDC), there are at least 476,000 people per year diagnosed with Lyme disease in the US alone (representing the incidence, not prevalence, of the disease) [3]. In the United States, the National Notifiable Disease Surveillance System (NNDSS) is responsible for sharing information regarding public health threats that qualify as “notifiable diseases”. Lyme disease is a recognized public health threat and is a designated notifiable disease. As such, Lyme disease is mandated to be reported by the CDC. Moreover, at least 14.5% of the world’s population may have had Lyme disease (and/or infection exposure), which is indicated by positive blood tests, with Central Europe, Eastern Asia, and Western Europe as the top three regions [7]. According to some researchers, the number is likely much higher as: (1) Many patients may be misdiagnosed with other severe and disabling conditions [8,9]; (2) The commonly used ELISA Lyme disease tests have a sensitivity averaging around 56% and check for an immune response to the *Borrelia*, which may not be present until at least a month after infection [10]; (3) According to researchers, only 20% [11] to 50–60% [12] of people with Lyme disease recall being bitten by a tick for several reasons, including the fact that ticks can be less than one millimeter and inject an analgesic into their host [12]; and/or (4) Per the authors’ hypothesis, patients with Lyme disease and CLD experience medical gaslighting, leading to misdiagnoses, delayed diagnosis, or a lack of diagnosis.

In this article, we ask: What are the full range of gaslighting techniques people with Lyme disease experience as they navigate the medical system? Are there demographic variables, such as geographical location, age, and sex, that correlate with a higher incidence of medical gaslighting?

Patients with Lyme disease often struggle to receive a timely diagnosis [13], leading to more deleterious health effects [9,14,15]. Compounding this issue are the vastly different treatments for Lyme disease (both acute and chronic). For example, the Infectious Disease Society of America’s (IDSA) treatment guidelines (updated in 2020) recommend 5–28 days of antibiotics depending on symptoms, followed by an additional 2–4-week course of IV antibiotics under certain circumstances [16]. In contrast, the International Lyme and Associated Diseases Society (ILADS) treatment guidelines recommend more individualized protocols based on patient responses [17]. According to ILADS, CLD is defined as multisystemic, “with symptoms and/or signs that are either continuously or intermittently present for a minimum of six months” [18] (p. 269). ILADS recognizes CLD as the result of an active and ongoing infection, which has either been untreated or previously treated [18]. Although not the focus of the current research, it is worth mentioning that tick-borne co-infections, which are not uncommonly found in persons with Lyme disease, greatly increase the challenges of both the diagnosis and treatment. The symptom complexes of these illnesses can overlap with those of CLD, and the treatment for the co-infections can require the use of different therapeutics than would suffice for Lyme disease alone [19,20].

Acute Lyme disease can include flu-like symptoms, extreme fatigue, headache, stiff neck, muscle soreness, joint pain, swollen lymph nodes, sore throat [21], and facial palsy [22]. The “bullseye rash” or erythema migrans (EM), which is considered to be the primary indicator of Lyme disease during the acute phase, is absent in most cases [12] or undetectable in others, particularly African Americans [23,24]. Further, only 6% [25] to approximately 20% [26,27] of Lyme rashes resemble a bullseye. This is unfortunate, as it is possible to cure Lyme disease in its acute phase [28,29,30] with at least 4–6 weeks of antibiotics, per the ILADS guidelines. After the initial IDSA-recommended length of antibiotic treatment for acute Lyme disease, 10–20% [9,31,32] to 36% of patients [33] will have ongoing symptoms.

*Borrelia burgdorferi* is a complex stealth pathogen, which can disseminate throughout the body and elude the immune response. Clinical studies show evidence of persistent *B. burgdorferi* infection in humans [34,35,36]. Although there are some overlapping symptoms of CLD and acute Lyme disease, including pain, polyradiculoneuropathy [37] fatigue, and sleep disturbance [38], CLD causes some symptoms that differentiate it from acute Lyme disease, including functional and structural brain abnormalities [39], cognitive impairment [13,40], neuroborreliosis [14], musculoskeletal and neurological morbidity, vision impairment [41], depression [9,42] (which can lead to suicide) [43], cardiac issues (including Lyme carditis) [44,45], gastrointestinal issues [46,47], joint pain, and Lyme arthritis [48,49]. The number of Lyme disease patients with lingering symptoms in the US was projected to be as high as 1,944,189 in 2020 [50]. Klempner [51] describes the quality of life for these patients as being equivalent to that of patients with congestive heart failure or osteoarthritis. Fallon and researchers [52] describe patients with Lyme encephalopathy reporting pain similar to post-surgery patients and fatigue similar to patients with multiple sclerosis. According to Johnson and researchers [53], CLD patients suffer a worse quality of life than people with multiple sclerosis and arthritis.

The World Health Organization ICD-11 issued new Lyme disease codes ratified by 194 nation members. The ICD-11 expanded to include severe and potentially fatal complications recognized in acute Lyme disease and/or CLD. The new ICD-11 codes for Lyme disease include nerve damage/degeneration, which can be verified by MRI, SPECT, and other tests; joint damage, which is verifiable by X-rays, ultrasound, etc.; inflammatory eye diseases; and heart issues such as rhythm irregularities and carditis, which can be diagnosed by EKGs, Holter monitors, and ultrasound.

Persistent infection has been documented in mice [54], dogs [55], and non-human primates [56,57,58,59]. In humans, positive culture and PCR results were found in synovium and synovial fluid specimens obtained from a patient 7 years after treatment [60], in an iris biopsy specimen obtained from a treated patient [61], and in DNA and via confocal microscopy in human spinal fluid and autopsy tissues (including the brain, heart, kidney, and liver) after extensive antibiotic treatment over a 16-year period [34]. In another study, the urine of 72 patients who were treated with antibiotics between 3 weeks and two months continually (with most retreated for 1–4 months after a few weeks pause) had positive PCR urine cultures [62]. Positive culture and PCR results were also found in the culture of blood, genital secretions, and a skin lesion of 12 patients despite 2–4 weeks of antibiotic treatment [63].

According to Dumes [64], contested illnesses are those that lack objective, biological markers and are differentiated from diseases that are medical conditions attributable to biological processes. The former lacks cultural legitimacy as any physical manifestations are deemed purely subjective. In this contestation is the chasm between the sufferers’ lived or embodied experiences and the cultural legitimation of their symptoms and suffering. As such, they are “illness[es] you have to fight to get” [65] because the patient must prove to medical practitioners that they indeed need medical attention, leading to “lengthy diagnostic odysseys” [66] (p. 2).

Many diseases were previously thought to be contested illnesses because their biological basis was not understood. As such, the onus was on the patient to actively prove to medical practitioners that they need medical attention because their symptoms have been delegitimized. This can lead to added stress and medical issues for the sufferer who is deprived of access to proper medical care [67]. Examples include inflammatory bowel disease [68], endometriosis [69], peptic ulcers [70], and, more recently, Long COVID [66]. Lyme disease and CLD remain contested [71], even though they are caused by a known pathogen. With regard to CLD, various detection methods, including histopathological and molecular testing and microscopy, immunoelectron microscopy [63], and polymerase chain reaction (PCR) [72], have provided evidence of infection from patients with persistent Lyme disease symptoms following antibiotic treatment. Uninfected ticks were also infected after placement on a previously treated symptomatic individual [32]. Despite this, CLD remains contested as well [64].

Medical gaslighting, which may be experienced by those with contested illnesses, can be defined as “a type of abuse aimed at making victims question their sanity as well as the veracity and legitimacy of their own perspectives and feelings” [73] (p. 4). That is, medical gaslighting is the outcome of viewing the patient’s concern as subjective and not attributable to an objective, biological cause. This can occur even if the practitioner does not consciously intend to gaslight the patient [73]. Medical contexts, which are characterized by a power imbalance between doctor and patient, can lead to a tendency to view the latter as incapable of accurately relaying their own symptoms to the practitioner [66]. Sebring [74] explains that medical gaslighting is evident when patients feel their concerns about their health have been dismissed. As Dumit [65] (p. 577) states, “Doctors, government, and insurance agencies appear to patients to be unable to hear their claims, denying them a social sick role and rendering them ‘just plain crazy’”. Research has shown that women and people of color are most likely to be targets of medical gaslighting [74].

Some examples of medical gaslighting that we also employ in our research are reflected in the peer-reviewed literature and include having concerns about health dismissed [74], being told that symptoms are psychosomatic or attributed to anxiety [66], the downplaying of pain [75], refusal to order patients imaging/lab work, and having symptoms attributed solely to “poor nutrition, mental health, lack of exercise, or obesity” [76] (p. 2). The authors of the current research would add that attributing symptoms solely to the aging process, dismissing patients’ bloodwork results, and outward manifestations of a medical condition (e.g., the EM rash) are indicative of medical gaslighting.

## 2. Materials and Methods

Data for the analysis were drawn from an original 46-question, anonymous, online survey hosted by Qualtrics and developed by the first author with input from Kristina Bauer and Jenna Luché-Thayer. The survey included a consent form and was reviewed and approved by Lamar University’s Institutional Review Board (IRB Number: FY23-28). It was posted in national and international patient-led online groups and Lyme disease non-profit organizations from 12 October 2022 to 9 December 2022. Our focus was on online groups as these forums have been an important tool for Lyme disease and CLD patients (e.g., they learn how to navigate the medical system and gain access to peer-reviewed publications). Such groups provide tools and references that support these patients becoming “lay experts of medical science” [66] (p. 3). Only respondents 18 years of age and older who identify as having Lyme disease/CLD or having minor children with Lyme disease/CLD were permitted to complete the survey. As delineated below, the majority of respondents were diagnosed via bloodwork ordered by a physician.

Respondents were asked a series of questions to determine their overall experiences with the medical community while they sought a diagnosis of, and treatment for, their Lyme disease and CLD. The survey measured demographic variables and experiences of medical gaslighting. For three questions, respondents used a slider, with endpoints of 0 and 100, to indicate their answers. Two questions measured how many years after symptom onset, and how many medical practitioners were seen, until the respondent received a Lyme diagnosis. The other question asked how many years the respondent had Lyme disease. For 12 questions, respondents used a slider, with endpoints of 0 and 50, to indicate how many medical practitioners (if any) had subjected them to different behaviors described as medical gaslighting in the peer-reviewed literature. The remaining questions had a multiple-choice answer format. The survey was administered in English.

Although we obtained data from respondents across the globe, the primary purpose of this project was to examine and analyze medical gaslighting experiences of US Lyme disease and CLD patients. Some data representing international contexts were included when the results were noteworthy (e.g., different from patterns observed in the US) or relevant to the research question under consideration (e.g., comparing experiences in other nations to the US).

## 3. Results

The final sample consisted of 986 respondents from 28 different countries, with the majority (95%) residing in one of five nations: the United States (n = 474, 48%), Australia (n = 151, 15%), Canada (n = 144, 15%), Ireland (n = 86, 9%), or the UK (n = 82, 8%). The US sample included individuals from all US states except Alaska, Nebraska, South Dakota, and Wyoming. The entire sample consisted of 835 females (85%) and 141 males (15%). Of the remaining 10 respondents, 6 indicated they were non-binary and 4 declined to indicate their sex. The sample was predominantly White (n = 904, 92%), with two Black respondents, three Native Americans, and three Asians. Of the remaining 72 respondents, 62 indicated their race was “other”, and 10 declined to answer.

When classified according to education level, 58 (6%) respondents had some high school education or less, 87 (9%) had a high school diploma or GED, 188 (19%) had completed some college, 109 (11%) had an associates or technical degree, 298 (30%) had earned a bachelor’s degree, 215 (22%) had earned a graduate or professional degree, and 31 (3%) declined to indicate their education level.

The sample consisted of 32 (3%) respondents who answered on behalf of their minor children under 18 years old, 36 (4%) respondents between 18 and 24 years old, 95 (10%) respondents between 25 and 34 years old, 200 (20%) respondents between 35 and 44 years old, 278 (28%) respondents between 45 and 54 years old, 213 (22%) respondents between 55 and 64 years old, 126 (13%) respondents who were at least 65 years old, and 6 (<0.01%) who declined to indicate their age.

Among the entire sample, 707 (71.8%) respondents identified as having Lyme disease based on a positive blood test result ordered by a physician, 132 (13.4%) diagnoses were based on a physician’s symptom-driven clinical diagnosis, 39 (4.0%) were based on a self-diagnosis following unspecified testing, and 19 (1.9%) were based on a symptom-driven self-diagnosis. An additional 85 (8.6%) indicated “other” as the basis for their Lyme disease patient identification, and 4 (0.3%) did not respond to this question. Of the 474 US respondents, 362 (76.4%) based their Lyme disease patient status on a positive blood test result ordered by a physician, 61 (12.9%) based it on a physician’s symptom-driven clinical diagnosis, 11 (2.3%) based it on a self-diagnosis following unspecified testing, and 7 (1.5%) based it on a symptom-driven self-diagnosis. From the US sample, 32 (6.8%) based their Lyme disease patient status on “other,” and 1 (0.2%) did not respond to this question.

The descriptive statistics for responses to medical gaslighting questions measured on a continuous scale (e.g., 0 to 50 or 100), for the entire sample, are presented in Table 1. In general, the standard deviation values indicate a large amount of variability in responses. Each of the response distributions is also positively skewed, meaning there are more responses at the lower end of the response scale than in a normal distribution. This is clearly indicated by each distribution mean exceeding the median, as well as the large difference between the upper quartile value and the maximum response value obtained. For these reasons, the mean of each distribution should be viewed with caution. It is probably best to consider median responses, rather than the mean, when attempting to understand the experiences of the average respondent.

The responses offer some insight into the relative frequency that these patients experienced various gaslighting behaviors. Rather than attending to the symptoms, patients were much more likely to be told by practitioners that they were just overreacting to their symptoms, there was no such thing as CLD, or that their symptoms were caused by normal aging, mental illness, or stress. Many patients also felt that medical professionals frequently implied the patient’s symptoms were merely psychosomatic.

An example of medical gaslighting is doctors not believing patients have Lyme disease even after a positive blood test result. Out of 474 total US respondents, 429 (90%) indicated whether they were diagnosed with Lyme disease via blood tests. We performed a Chi-square test of independence to determine whether a positive blood test influenced a doctor’s belief the patient had Lyme disease. The analysis revealed that a patient’s positive blood test status did not influence how likely a doctor was to believe the patient had Lyme disease, χ^2^ (1, n = 429) = 1.09, *p* = 0.30. Doctors were no more likely to believe a positive blood test patient had Lyme disease (79% unconvinced) than a patient who had not obtained a blood test (74% unconvinced).

Medicine is still an industry with a sex imbalance favoring males [77]. Although this trend is changing, women are still underrepresented in medicine [78]. The overrepresentation of male medical professionals and the fact that patients with Lyme disease may need to self-advocate more often than the typical patient make it plausible that medical outcomes for female patients may be systematically different from male patient outcomes. To explore this, we categorized US respondents based on their sex, whether they had asked for a Lyme disease test, and if their doctor had refused or granted their request. We then conducted two separate Chi-square tests of independence. The first analysis revealed that a patient’s sex did not influence how likely the patient was to request a Lyme disease test, χ^2^ (1, n = 466) = 1.73, *p* = 0.19. Female patients were just as likely (72%) as their male counterparts (80%) to request a Lyme disease test. The second analysis revealed that a patient’s sex did not influence how likely the doctor was to refuse the patient’s request, χ^2^ (1, n = 340) = 0.04, *p* = 0.84. Doctors were just as likely to refuse a male patient’s test request (60%) as a female patient’s request (59%). However, it must be noted that women participants were vastly overrepresented in our sample. Hence, these findings are not necessarily indicative of a lack of a sex disparity.

Another variable that may affect medical outcomes for these patients is where they live and seek medical care. The CDC classifies 15 states, and the District of Columbia, as having a “high incidence” of Lyme disease [2]. Based on this designation, we coded respondents living in these states as residents of a Lyme endemic (LE) state. A total of 189 (40%) respondents reported living in an LE state. Does living in a place where Lyme disease is more common affect these patients’ experience while interacting with the medical community? To explore this question, we conducted a series of Chi-square analyses of independence (for categorical dependent variables (DVs), see Table 2) and Mann–Whitney U tests (for continuous DVs, see Table 3) using Lyme endemic state status (LE or non-LE) as the predictor variable. Although one would normally use an independent sample *t*-test to compare LE and non-LE residents on continuous variables, there was not sufficient homogeneity of variance between the compared distributions for nearly all the DVs. Therefore, the nonparametric Mann–Whitney U test was necessary.

Results indicated that residing in an LE state does seem to influence some experiences patients had while seeking testing and treatment (see Table 2). Doctors in LE states were more likely to grant the patient’s request for a Lyme disease test (49%) than doctors in non-LE states (35%), χ^2^ (1, n = 345) = 7.10, *p* = 0.008. Doctors in LE states were also more likely to believe a positive test result (28%) than doctors in non-LE states (18%), χ^2^ (1, n = 432) = 6.62, *p* = 0.01. These two findings were corroborated by LE state patients’ reports that they generally obtained a Lyme disease diagnosis in fewer years (M = 10.58, SD = 12.87) and after seeing fewer doctors (M = 11.51, SD = 12.86) than those living in non-LE states (M = 14.51 years, SD = 14.51; M = 15.77 doctors, SD = 16.10).

Geography also influenced how doctors explained patients’ Lyme disease symptoms (see Table 3). Patients in non-LE states encountered more doctors (M = 7.09, SD = 9.95) than patients in LE states (M = 2.35, SD = 4.78) who told them they could not possibly have Lyme disease because there were no ticks or Lyme disease in the area. A greater number of doctors in non-LE states (M = 3.99, SD = 9.15) than in LE states (M = 2.07, SD = 6.72) were also more likely to tell patients they did not have Lyme disease despite the presence of the telltale “bullseye” rash. Finally, there was a marginally significant (*p* = 0.053) result showing more non-LE state doctors (M = 6.96, SD = 9.97) than LE state doctors (M = 5.27, SD = 8.39) directly told patients their Lyme disease symptoms were just in their head (i.e., psychosomatic).

We also wanted to determine whether the country a person resided in was associated with these patients’ medical outcomes. We examined whether the country of residence predicted a doctor’s willingness to test for Lyme disease if a patient requested it, whether a doctor believed a positive Lyme test result, or whether a doctor asserted the patient did not have Lyme disease despite having a bullseye rash. For all categorical DVs, we used Chi-square tests of independence, and for all continuous DVs, we used Kruskal–Wallis paired-rank tests because of large differences in sample sizes and sample variances. For all analyses, data were limited to the five countries with sufficient sample sizes (i.e., US, Canada, UK, Ireland, and Australia). The following countries were also represented, but there were fewer than 10 respondents in each: Romania, New Zealand, Germany, Mexico, the Netherlands, Spain, Switzerland, Finland, Hungary, South Africa, Sweden, Belgium, Bosnia and Herzegovina, Brazil, Bulgaria, Denmark, Egypt, France, Norway, South Korea, Turkey, United Arab Emirates, and Vietnam.

Results showed that country was associated with how often a doctor granted a patient’s request for a Lyme disease test, χ^2^ (4, n = 673) = 16.89, *p* = 0.002. As seen in Table 4, Australian doctors were less likely to grant the request for a test (19%) than the average doctor across the other four countries (38%). The country of residence was also associated with how often a positive test result convinced doctors the patient had Lyme disease, χ^2^ (4, n = 863) = 26.89, *p* < 0.001. Australian doctors believed a positive test result less often (6%) than the average doctor (17%). Interestingly, doctors in the US (22%) and Ireland (23%) believed Lyme disease test results slightly more often than average. More Australian doctors (M = 6.21, SD = 11.04) told their patients they did not have Lyme disease despite the patient exhibiting the bullseye rash normally associated with Lyme disease, H(4) = 15.48, *p* = 0.004. Follow-up Dunn tests comparing mean rank scores for all possible country pairings showed only US doctors were statistically less likely (M = 3.24, SD = 8.37) to tell patients they did not have Lyme disease despite their bullseye rash, z = 2.93, *p* < 0.05.

## 4. Discussion

Interactions with doctors who doubt the lived experiences of patients with Lyme disease have health implications (e.g., delayed treatment can lead to chronicity) and psychological ramifications. Most illustrative of the textbook definition of medical gaslighting, a striking majority of respondents felt that they were treated as a marginalized patient group, they were told by practitioners that they were overreacting, that there is no such thing as CLD (71.8% in the entire sample, 68.6% in the US sample), or that their symptoms were caused by normal aging, mental illness, or stress. We also contend that a median of 10 doctors seen before diagnosis is, in and of itself, highly suggestive that medical gaslighting occurred. Adrion and researchers [79] indicate that five physicians were seen by the average Lyme disease patient prior to proper diagnosis, and Johnson and researchers [80] glean that half of their 2424 respondents saw at least seven physicians prior to their Lyme disease diagnosis. Medical gaslighting contributes to the scope of this public health threat being underrealized, underreported, and under-addressed.

What is most interesting—and troubling—is that proof of an ongoing Lyme disease infection via bloodwork would seem to necessarily situate it outside the parameters of a contested illness. However, according to our data, many with positive blood tests still faced medical gaslighting as if they indeed have a contested illness. According to our data, even the telltale bullseye rash left many doctors unconvinced of active Lyme disease infection (n = 527).

According to Davis [81], the long-term effects of medical gaslighting include anxiety, depression, PTSD symptoms, and trauma. This becomes a vicious cycle as the psychological symptoms can exacerbate physical symptoms. Furthermore, doctors who suggest, either directly or indirectly, that Lyme disease patients’ physical pain is attributable to psychological issues (e.g., somaticized depression or anxiety), may cause, or at least exacerbate, those very issues. 

## 5. Conclusions

Medical gaslighting does not occur in a vacuum. The contestation of CLD has created a climate in which doctors may be less inclined to believe that Lyme disease patients’ persistent symptoms are attributable to an ongoing infection. Thus, such patients may not receive treatments for their underlying infection. We find it noteworthy that our data gleaned such a high incidence of medical gaslighting despite increased awareness about Lyme disease and CLD over the decades. That is, the availability of data is incommensurate with the treatment of patients with Lyme disease and CLD.

Our study is not without its limitations. People of color were essentially absent from our sample. There are several possible reasons for this: (1) As previously mentioned, the EM rash may present differently on some people of color, leading to a lower detection rate [23,24]. (2) There may be a lower incidence rate for people of color. Per Adekoya [82], “Incidence rate for Lyme disease is approximately 11 times greater for Whites than it is for African Americans”, which may be due, in part, to risk exposure due to geographic variables. (3) Lack of access to care due to racial and ethnic disparities [23]. (4) The higher likelihood of people of color being medically gaslit [74]. (5) The perception that patient-led groups are White spaces and/or the proliferation of online racism [83]. Further, recent South American immigrants to Lyme-endemic US states like NY [84] did not fully appreciate the danger that ticks/Lyme disease posed and were not well-versed in recognizing symptoms. People of lower socioeconomic status may lack access to the technology to take part in the research and might also have a particularly difficult time accessing the medical specialists to address Lyme disease symptoms. The fact that our survey was only available in English is yet another limitation of our study.

Considering the limitations of this work, further research on the experiences of Lyme disease patients is desperately needed. There are numerous viable avenues of inquiry. What are the experiences of people of color with Lyme disease? Does changing roles from provider to patient when a medical practitioner is diagnosed with Lyme disease alter healthcare professionals’ perceptions of medical gaslighting? Mounting evidence of persistent infection underscores that patients with Lyme disease are deserving of serious consideration by medical practitioners. The Hippocratic oath demands nothing less.

## Figures and Tables

**Table 1:** Descriptive statistics for experiences with Lyme disease and medical community.

Question	N	Mean	Median	SD *	LQ *	UQ *	Max *
Years since infection until diagnosis	952	11.52	7	11.86	3	17	66.00
Doctors seen until diagnosis	958	13.94	10	14.89	5	17	100.00
Years you have had Lyme disease	962	17.31	14	13.53	7	25	75.00
Told overreacting to symptoms ^1^	925	9.26	6	10.12	3	11	50.00
Told symptoms just normal aging	825	7.32	4	9.29	2	10	50.00
Told not Lyme, no ticks in your area	793	6.85	3	9.88	1	8	50.00
Told not Lyme because no rash	700	5.73	3	8.69	0	8	50.00
Told not Lyme despite bullseye rash	527	3.94	0	8.62	0	4	50.00
Told no such thing as CLD	825	7.61	4	9.96	2	10	50.00
Told symptoms from being overweight	539	3.87	1	7.78	0	5	50.00
Told change diet to end symptoms	582	4.15	2	7.32	0	5	50.00
Implied symptoms psychosomatic	895	8.19	5	9.89	2	10	50.00
Told symptoms psychosomatic	803	6.60	3	9.71	1	7	50.00
Told symptoms are from mental illness	850	7.66	4	9.80	2	10	50.00
Told symptoms due to stress	802	7.48	4	10.30	2	9	50.00

^1^ From this entry onward, responses indicate the number of medical professionals encountered who interacted with the respondent in the manner described. * SD: standard deviation; LQ: lower quartile; UQ: upper quartile; Max: greatest value response provided.

**Table 2:** Frequencies and Chi-square results for medical outcomes based on state classification.

Question	LE State		Non-LE State		χ^2^ (1)	*p*
	Yes	No	Yes	No		
Doctor refused to administer Lyme test	73	70	130	69	7.10	0.008
Doctor assumed you were a drug seeker	108	55	160	91	0.35	0.555
Diagnosed with conversion disorder	10	131	14	203	0.01	0.935
Diagnosed with Munchausen syndrome	8	165	6	253	1.77	0.183
Doctor still unconvinced after Lyme diagnosis	123	48	212	46	6.62	0.010
Treated you as marginalized patient group	158	13	238	16	0.31	0.579
Misdiagnosed with autoimmune disease	88	91	156	119	2.17	0.141
Told not to use antimicrobials/antibiotics	94	51	119	81	0.66	0.416
Condition suffered from inadequate treatment	171	7	262	10	0.03	0.871
Condition suffered from no early treatment	171	6	269	10	0.01	0.920
Child’s condition suffered misdiagnosis	34	18	69	19	2.98	0.084
Child’s health suffered from lack early treatment	35	7	74	10	0.58	0.445
Doctor said no such thing as gestational Lyme	49	43	93	61	1.37	0.243

**Table 3:** Mann–Whitney U test results for medical outcomes based on state classification.

Question	LE State		Non-LE State		z	*p*
	N	Mean Rank	N	Mean Rank		
Years since infection until diagnosis	185	202.69	272	246.90	3.51	<0.001
Doctors seen until diagnosis	184	207.63	279	248.08	3.18	0.001
Told overreacting to symptoms ^1^	177	211.04	264	227.68	1.34	0.179
Told symptoms just normal aging	155	188.74	241	204.78	1.36	0.174
Told not Lyme, no ticks in your area	118	124.79	232	201.29	6.69	<0.001
Told not Lyme because no rash	141	169.69	212	181.86	1.10	0.272
Told not Lyme despite bullseye rash	97	110.54	149	131.94	2.30	0.021
Told no such thing as CLD	155	188.13	233	198.74	0.91	0.361
Told symptoms from being overweight	103	129.60	161	134.36	0.49	0.622
Told change diet to end symptoms	118	134.93	172	152.75	1.78	0.076
Implied symptoms psychosomatic	166	203.13	260	220.12	1.39	0.165
Told symptoms psychosomatic	145	171.65	224	193.64	1.93	0.053
Told symptoms are from mental illness	154	197.94	251	206.10	0.68	0.496
Told symptoms due to stress	149	183.20	237	199.97	1.44	0.151

^1^ From this entry onward, responses indicate the number of medical professionals encountered who interacted with the respondent in the manner described.

**Table 4:** Frequencies and Chi-square results for medical outcomes based on country.

Question	Country	Yes	No	χ^2^ (4)	*p*
Doctor(s) refused to test for Lyme?	United States	203	139	16.89	0.002
	Australia	68	16		
	Canada Ireland United Kingdom	73 35 38	41 28 32		
Doctor(s) still unconvinced by test result?	United States	335	94	26.89	<0.001
	Australia Canada Ireland United Kingdom	135 116 62 69	8 16 19 9		

## Data Availability

The data presented in this study are available on request from the second author. The data are not publicly available because the authors are currently preparing other publications using the same data set.
